# The mechanism and dynamics of the sedimentation of styrene/divinylbenzene microplastics from water and the solidification of the precipitate with an alum-based coagulant “BUCOCHEM”

**DOI:** 10.1371/journal.pone.0346838

**Published:** 2026-07-29

**Authors:** Vasyl Kulish, Sergiy Boruk, Igor Winkler

**Affiliations:** 1 Department of Chemistry and Food Quality Assessment, Institute of Biology, Chemistry and Bioresources, Yuriy Fedkovych National University of Chernivtsi, Chernivtsi, Ukraine; 2 Department of Medicinal and Pharmaceutical Chemistry, Bucovinian State Medical University, Chernivtsi, Ukraine; National Research and Innovation Agency, INDONESIA

## Abstract

Although publications on the efficiency of various coagulants in removing microplastic contamination from water are quite abundant, detailed information on the coagulation/sedimentation mechanisms and dynamics, and on the formation of microplastic particle flocs, is less common. In this article, the mechanism and dynamics of coagulation, flocs formation, and sedimentation of styrene/divinylbenzene microplastic particles sized between 400 and 500 µm from water with the use of an alum-based coagulant “BUCOCHEM” have been investigated. The research has been conducted using jar tests to study coagulation/sedimentation dynamics and visual microscopic observations to examine floc formation. It was found that this coagulant promotes better sedimentation and compaction of the plastic particles, ensuring the formation of a dense, more stable, and easily removable precipitate layer. A 0.1 wt % of the coagulant leads to a decrease in the height of the polymer sediment layer by 14% after 48 hours, as compared to the coagulant-free sedimentation. Furthermore, the former sediment appears more compact and stable. Even the lower 0.05–0.07 wt% of BUCOCHEM ensures the formation of three-dimensional flocs, which sediment into a compact and stable precipitate layer, effectively resisting secondary resuspension. Although such concentrations of BUCOCHEM lead to some decrease in the sedimentation rate (from 3.5 to 2.5–2.7 mm/s), it is more important that the resulting precipitate layer becomes more compact and stable against secondary resuspension than without the coagulant. The process of the precipitate layer compacting and solidification is mostly complete within 24 h. These results indicate the most optimal concentration of BUCOCHEM (0.05–0.07 wt%) and seem useful for potential application of this coagulant to treat water/wastewater for the removal of microplastic contamination.

## Introduction

Plastic particles are quite stable and persistent in the environment, and their natural decomposition in water lasts from several months to decades. It should also be mentioned that once a plastic pollutant enters water, it often breaks down into fragmented microplastic particles, which increases its environmental harmfulness [1–3].

Since “microplastics” are artificial materials with particles sized between 1 µm and 5 mm, they can actively adsorb other pollutants on the surface, which increases their negative environmental impact. It should be understood that microplastics can enter the environment in the form of small particles originating from various additions to consumer goods (granules, fibers, etc. added to cosmetics, housekeeping chemistry, and other items) or it can appear as a result of partial decomposition of bigger plastic items like plastic bags, other packaging, details of various machines and mechanisms, etc.

Microplastic (MP) pollution in the environment has numerous adverse effects and has been detected in all aquatic environments, including seas, oceans, rivers, lakes, and even groundwater, as well as in water sources used for the production of drinking and technical water [4–7].

Various classical water cleaning technologies can be applied to remove MP particles, including filtration/sedimentation alone, coagulation/flocculation followed by filtration/sedimentation/floatation, and others. However, their cleaning effectiveness critically depends on the size of the particles to be eliminated, and it rapidly deteriorates as the particle size decreases. Therefore, aggregation of MP particles seems reasonable in the context of improving their removal from water.

The use of various alum, PAC, and ferric-based coagulants – either alone or combined with flocculants – significantly enhances the efficiency of removal of microplastic, particularly for weathered plastic particles. Commonly, the removal of smaller and weathered particles is better than that of greater and intact particles, while in the context of coagulants’ composition, alum/PAC/Al_2_(SO_4_)_3_-based coagulants show a higher performance than the ferric-based agents [2,3,8,9]. As reported in [2], alum and PAC coagulants demonstrate similar performance in removing various pristine MP particles. Its efficiency ranged between 65 and 82%, depending on MP composition. In the case of weathered MP, it increased, for some types of MP, up to 98–99%. The authors of [3] also reported the highest coagulation efficiency of alum and aluminum sulfate-based agents, which reached 100% for some types of polyethylene MP particles. Ferric-based coagulants can also ensure such performance, but at a sufficiently higher dosage. As found in [8,9], removal performance for polystyrene-based MP was lower than that for polyethylene particles, while PAC was still more effective than ferric, and the removal of smaller particles was more complete than in the case of larger MPs.

Although MP coagulation/precipitation can be efficient, secondary resuspension of the primary precipitate layer can seriously reduce the overall effectiveness of water and wastewater cleaning technologies. As reported in [10], even weak turbulence significantly increases the concentration of MP particles by causing resuspension of the preliminary settled precipitate. Piping, mixing, and other operations in water/wastewater treatment processes create stronger turbulence, making precipitate stability against secondary resuspension critically important. This article examines this issue for the coagulant BUCOCHEM.

To ensure high resistance of the precipitate to secondary resuspension, it must solidify by developing an internal matrix or structure. For instance, the authors of [11] found that chemically pretreated MP particles, which have a more weathered, porous, and corroded surface, form larger flocs with a greater fractal dimension. These larger, branched flocs can cohere more effectively and form more stable aggregates. Even when mechanically disrupted by stirring or other external forces, their recovery is quicker and more effective than that of smaller flocs formed from untreated particles with smoother surfaces. Thus, if BUCOCHEM promotes the formation of larger, more branched MP flocs, these structures are likely to show greater resistance to secondary resuspension than smaller, more linear particles.

It should be noted that pH significantly affects various parameters governing aggregation, coagulation, and sedimentation of MPs, and has been extensively studied [3,8,9,12]. However, the influence of pH has not been investigated in our study, as the coagulant under study is intended for use with large volumes of wastewater, where pH correction is typically not performed. Given that the pH of regular wastewater typically ranges between 5.5 and 7.5, the direct addition of the coagulant to the treated water and the selection of the most effective dosage are usually considered more technologically appropriate for MP removal. Therefore, in this study, pH was left uncorrected, at its natural level.

In this context, it seemed interesting to investigate and compare the coagulation dynamics and mechanism for removing MP particles from water using the coagulant BUCOCHEM, as well as the ability of the precipitate to resist secondary resuspension, which can reduce overall water-cleaning efficiency.

## Materials and Experimental Methods

This investigation was conducted on a model disperse system made of the ground particles of Copolymer-8B (a copolymer of styrene and divinylbenzene, PS-DVB) and distilled water. An alum-based reagent BUCOCHEM by LLC “Modern Environmental Technologies” (Ukraine), produced in 2023, lot number 09102023, was used as a coagulant. The technical specifications of the polymer and coagulant are given in Tables 1 and 2.

**Table 1:** Technical specification of Copolymer-8B.

Parameter	Value
Appearance	Inert spherical colorless grains
Granulometric composition, µm	400 ÷ 800
Mass fraction of the polymer, %	> 95
Bulk weight, g/cm^3^	0.55–0.7
Genuine density, g/cm^3^	1.05–1.07
Water content, %	< 1.0

**Table 2:** Technical specification of BUCOCHEM.

Parameter	Value
Appearance	Colorless or light yellowish-greenish liquid
Mass content of Al, %	7.47–8.48
Mass content of Al_2_O_3_, %	14.1–16.0
Relative basicity, %	56–75
Insoluble residue, wt%	< 0.1
Density, g/cm^3^	1.15–1.39
Mass content of Fe, %	< 0.05
Mass content of Cl^-^, %	8- 22
pH	1.5–4.0
Viscosity at 20 °C, mPa*s	10–65

### Sieving the MP fraction, evaluation of the size and shape of single and aggregated particles

A 400−500 µm fraction of Copolymer-8B has been sieved out and separated for further investigation. The size, shape, and configuration of the particles and/or their aggregates were evaluated visually using an MBS-9 microscope. This MP fraction was selected because it is primarily formed during routine bulk handling and transfer of this polymer, contaminating wash-offs and industrial wastewater.

### A method of introducing of the coagulant to the water samples and the sedimentation conditions

The coagulant has been added to the water samples as a 3−5% aqueous solution. The system was stirred thoroughly immediately after the addition of the required amount of coagulant for 20−30 seconds using a mechanical mixer at 1−2 s^-1^. The moment of stirring completion was considered the start of sedimentation.

Also, we did not correct the chemical composition of the water samples. Although this parameter is quite influential, real water/wastewater purification technologies rarely use preliminary treatment or correction of the water composition, except for heavily contaminated industrial wastewater. On the contrary, the water temperature must be kept under 60 °C. Otherwise, the process of coagulation and sedimentation can turn unpredictable, splitting into local subprocesses even before a uniform coagulation distribution across the system is reached. That is why the temperature of all samples was kept at 20–25 °C.

### Sedimentation and compacting of MP particles

The jar test was conducted to compare the initial settling rate and to observe formation, hydrophilization, and further compacting of the sediment layer. A 5 g sample of the polymer was poured into a glass beaker and mixed with 10 mL of distilled water. The mixture was stirred for approximately 1 minute, until it appeared visually uniform, and then transferred to a narrow and tall graduated glass cylinder up to the zero mark.

The dispersion phase of a just-prepared mixture consisted of a single granulometric fraction sized 400–500 µm, without coagulated flocs, which require some time to form. That is why, a distinct visible margin between the sediment layer and the transparent liquid formed within 10–20 seconds. Then, this margin descended until the primary stabilization of the sediment layer occurred.

The rate of sedimentation was determined as


$$\begin{array}{c} W=\frac{h}{\tau }, \end{array}$$ (1)

where *h* is the height of the clarified liquid layer above the sediment (mm), measured over time τ, s. To minimize individual reading errors, the values of *h* was determined by five independent observers, and then their readings were averaged.

After 2–3 minutes of initial settling, the suspension separated clearly into a lower sediment layer and an upper transparent liquid layer. In all experiments, the initial height of the sediment layer was 6.9 cm at 20 °C.

### Determination of hydrophilization and compacting of the polymer suspension

After initial settling, the MP suspension layer underwent hydrophilization and secondary compaction resulting in changes in its structure and volume/height. These changes were evaluated at 0.5, 6, 24, and 48 hours after initial settling. All experiments were repeated 7–10 times and then they results were averaged.

### Sampling the polymer particles for microscopic examination

When needed, a thin glass microprobe was used to take a sample of the polymer suspension from the middle of its phase. Then, a sample was placed on the microscope slide, covered, and examined visually.

## Results and discussion

Even without a coagulant, the surface of MP particles is getting slightly hydrophilized, forming a hydrate layer surrounding each particle. This “false hydrophilization” of a totally hydrophobic surface occurs due to the residual surfactants/emulsifiers stuck in the bulk of the polymer during its synthesis. As these residual compounds are mostly ionic, they facilitate some secondary hydrophilization of the plastic surface [13].

Such hydrophilized particles can aggregate, forming globular ensembles that capture some water in the interparticle space, resulting in some swelling of the sediment layer. This process is rather slow, as the amount of the residual surfactants/emulsifiers is low.

The thin, translucent hydrate layer and the globular, aggregated MP ensembles were observed microscopically in the samples taken from the sediment layer. Over time, such aggregated particles are getting compacted, reducing the interparticle distance and forcing the trapped water out of the interparticle spaces, which results in an overall decrease in the height and volume of the sediment layer.

It should also be mentioned that not all particles are getting hydrophilized. Perhaps, those consisting of a very low amount of the residual surfactants/emulsifier in the surface layer remain hydrophobic. As a result, they do not coagulate and settle individually.

**Fig 1:**
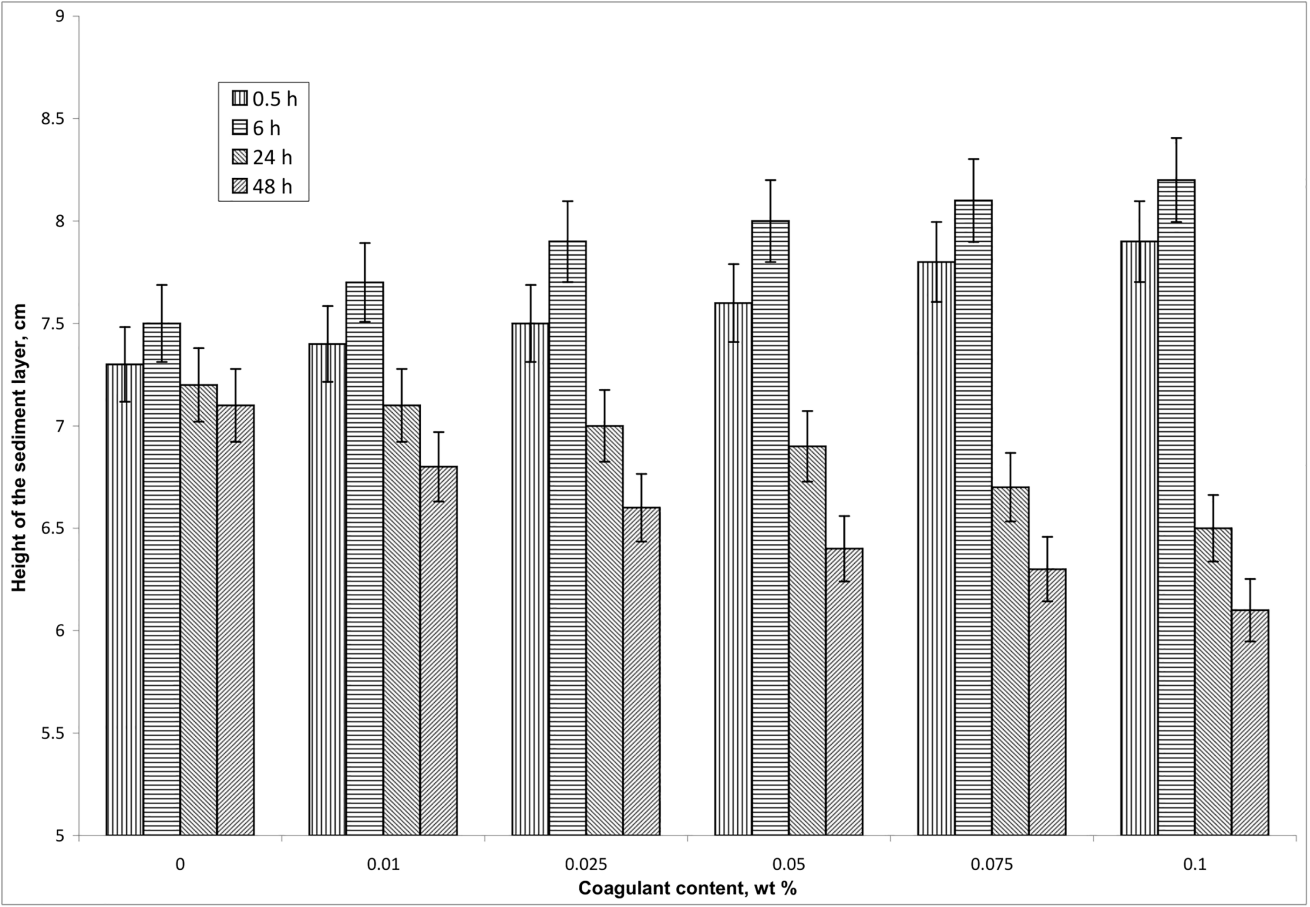
Heights of the polymer sediment layer without/with a coagulant admixture after 0.5, 6, 24, and 48 hours. The relative error of the sediment layer height determination was 2.5%.

**Fig 2:**
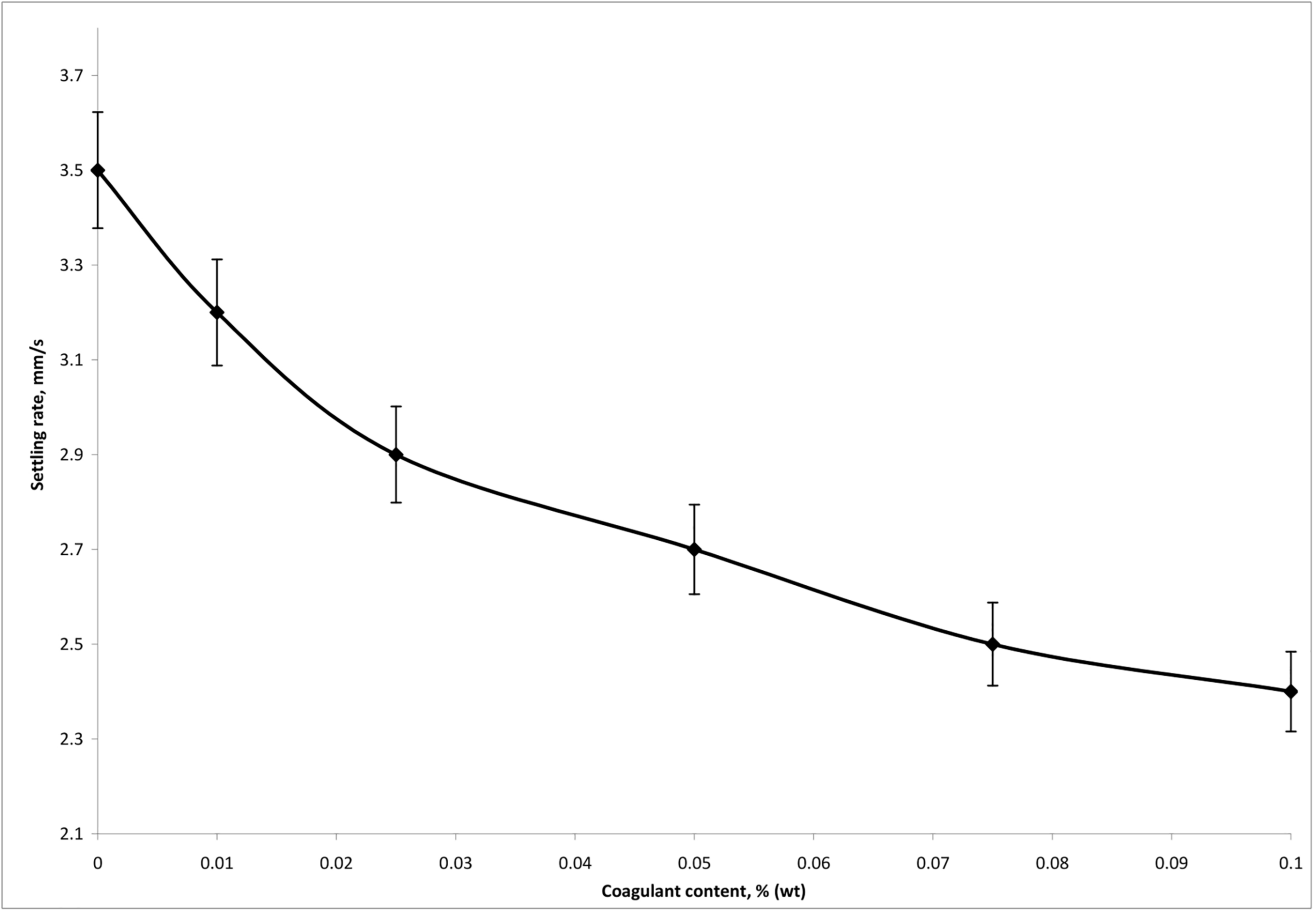
Dependence of the rate of settling of the MP particles on the coagulant content. The relative error of determination was 3.5%.

Adding a coagulant to the system intensifies all these processes, as this agent promotes better hydrophilization of the particles. As a result, more intense and rapid hydrophilization, and a subsequent deeper compacting of the sediment layer occur. These processes are represented in Fig 1. As seen in this Fig, increasing the amount of coagulant in the system causes the formation of a thicker sediment layer and improves the removal efficiency of microplastics (MPs). Although the sedimentation rate decreases slightly with higher coagulant concentrations (see Fig 2), this effect is minimal and does not significantly extend the time needed for complete sedimentation and MP separation. When the coagulant concentration is below 0.075%, the resulting aggregated particles are relatively small. Since these particles appear more compact, they are more easily resuspended, making filtration and separation more complicated. In contrast, a coagulant concentration of 0.075% produces larger, less compact flocs that are less prone to resuspension, facilitating their removal from the water. Increasing the coagulant concentration beyond this range does not further enhance MP removal, as the removal percentage plateaus while the sediment becomes increasingly loose. Therefore, a coagulant concentration of 0.05–0.075% appears to be optimal for the removal of microplastics from water. This conclusion seems statistically relevant since even starting from 0.025%, the precipitate layer compacting between 6 and 24 h greatly exceeds both experimental error intervals (see Fig 1). After 0.075%, the final precipitate layer height does not change much, and that is why the coagulant’s concentration of 0.05–0.075% seems the most optimal.

As reported in [14], loose and linear aggregates of plastic particles are compacting more slowly than either non-aggregated individual particles or denser, globular aggregates. This finding agrees with our results, which demonstrate that aggregated MP ensembles shift from loose and linear to denser and more globular forms (see further results and discussion below). This structural rearrangement promotes more compact packing of the MP ensembles in the sediment layer, resulting in a decrease in its height and volume. Faster and more efficient sedimentation of globular, dense aggregates with higher fractal dimensions has also been observed in [15]. Similar processes of sediment compaction and regrowth have also been reported in [16] for low-density polystyrene MP beads.

The rate of settling also depends on the amount of coagulant added. The density of MP particles is only slightly higher than that of water (see Table 1). When a secondary hydration layer forms around a plastic particle, the density of this hydrophilized object decreases further, approaching that of water and slowing the settling. Furthermore, when loose and large flocs are formed, they encounter greater hydrodynamic resistance as they settle, leading to additional slowing of the process.

Without the addition of a coagulant, the dispersed particles sediment individually at a rate of more than 3 mm/s (Fig 2). They do not aggregate and can move comparatively freely, without the high hydrodynamic resistance of the water. It should also be noted that without a coagulant, the resulting sediment was rather dense and appeared to contain only a minimal amount of immobilized water. Also, agitation caused the sediment to disperse, but it settled rapidly after the agitation ceased. An initial height/volume of the sediment layer was restored within 5 min after agitation; however, the layer remained unstable, causing the system to become turbid even after minor shaking or disturbance.

Once a coagulant is added, the rate of settling decreases below 3 mm/s (Fig 2). Since a coagulant facilitates formation of larger and more loose aggregates, which encounter a higher hydrodynamic resistance, their rate of sedimentation slows down. On the other hand, in this case, the settlement forms a denser and more compact layer, and the external disturbance does not easily lead to its rising. As mentioned above, it is caused by a larger size of the coagulated flocs and an adhesion between aggregated particles that develops during the settling and formation of the sediment. This process is completed within 24 hours, after which the settlement solidifies into a single layer that does not break into separate particles or flocs upon regular stirring or shaking.

Similar results were reported in [17,18]: the authors observed the deceleration of the settling rate of various MP particles, depending on irregularities in their surface shape and/or aggregation. The more weathered or irregular the particle’s surface, or the more aggregated they are, the slower they would sediment. Particular settling rates reported in [17] were quite close to our results. The settling rate reported in [18] for polystyrene of a similar size was greater, but this difference could be caused by different polymer expansion grades and differences in the densities of our PS-DVB polymer and pure polystyrene used in [18].

It is important to note that the overall time required for nearly complete sedimentation, whether or not a coagulant is used, is relatively short. While our study did not specifically assess the completeness of MP removal, the authors of [16] reported that this can be accomplished within 30 minutes. Therefore, sediment compactness and resistance to secondary resuspension are key factors influencing the efficiency of MP removal from water. In this context, our results are promising, as even a low concentration of BUCOCHEM significantly stabilizes the sediment.

This stabilization occurs as a result of forming a three-dimensional network within the sediment layer. In the absence of a coagulant, the sediment can be easily resuspended even after 24 hours, breaking into individual particles or small aggregates that resettle separately. When a coagulant is used, the sediment remains stable and does not resuspend after shaking, remaining clearly separated from the liquid.

**Fig 3:**
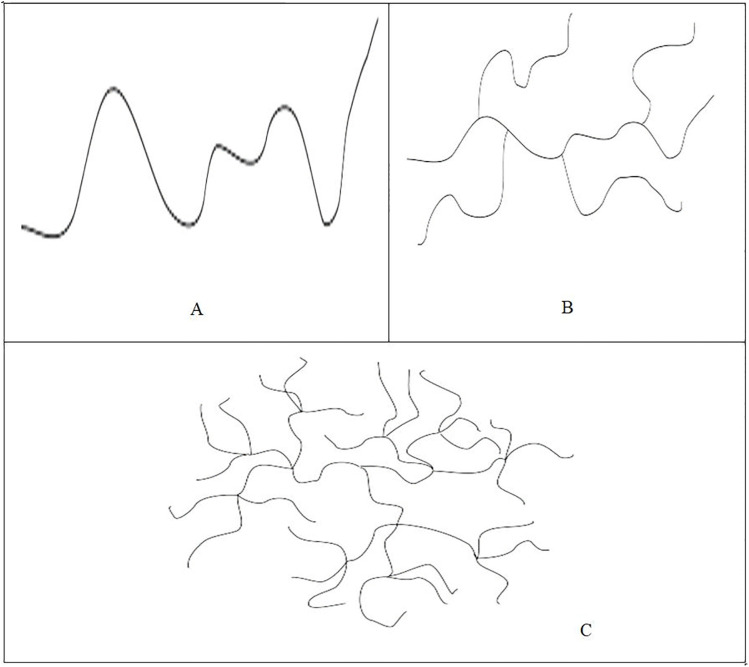
Spatial pattern of the sediment flocs: A – simple and linear (< 0.02% of a coagulant); B – longer linear with initial branching (0.02-0.05%); C – developed branching (>0.05%).

Microscopic observation of the sediment samples proved that the shape and pattern of the flocs changed depending on the coagulant content. As mentioned above, a coagulant promotes better hydrophilization of the surface of MP particles, leading to a more intense interparticle interaction. As a result, the particles can better aggregate and grow into longer, more branched, closely compacted, and more stable ensembles. Indeed, predominantly simple linear ensembles were observed in systems with low concentrations (below 0.02 wt% coagulant) (see Fig 3A). As the coagulant content increases, more complex, longer, and branched three-dimensional flocs form in the sediment (Fig 3B and3C). First, they remain separated (Fig 3B), but then bind together, forming massive aggregates that extend across the entire sediment layer (Fig 3C). This way, the flocs can efficiently interlace and aggregate together, forming a stable sediment that is easily removable and efficiently resists resuspension.

The most efficient concentration of BUCOCHEM was determined to be 0.05–0.075 wt%. At this range, relatively large three-dimensional flocs form and bind together into a stable sediment, which does not resuspend upon stirring. At lower concentrations, the forming flocs are smaller, and the sediment lacks sufficient stability against resuspension. Increasing the coagulant concentration above 0.075% does not provide additional stabilization of the sediment.

Similar processes are described in [19], where the authors discuss the details of alum-based floc formation, development, and compacting. Due to a combination of aluminum oxo-hydroxides formation, polymerization, and flocculation, the spatial geometry of the flocs changes from linear to planar and then to three-dimensional. The precipitate initially forms as an amorphous, bulky phase, which then undergoes compaction and solidification/crystallization —the same processes have also been observed in our experiments.

As reported in [20], branched three-dimensional flocs (like those shown in Fig 3, C) are highly effective at removing suspended particles. In addition to adsorption mechanisms, these flocs can mechanically capture and trap particles within the “caves” formed inside their bulk structure.

It should also be noted that the most effective water cleaning performance of BUCOCHEM, reached at 0.05–0.075 wt% of the coagulant, is comparatively competitive. Some other alum-based agents used in [19,21] have demonstrated the best cleaning efficiency with respect to natural turbidity of water at concentrations ranging between 0.01 and 0.05 wt%. Since natural turbidity of water is mostly caused by the clay and organic (algae) particles, which are either more hydrophilic and bear higher electric charge (clay) or rather larger (algae), our results featuring 0.05–0.075 wt% as the most optimal content of BUCOCHEM for the removal of lower charged and more hydrophobic MP, seem fair.

## Conclusion

The addition of an alum-based coagulant BUCOCHEM leads to the hydrophilization of the surface of PS-DVB MP particles 400–500 µm in size. These hydrophilized particles aggregate more readily, coagulate, and form large, branched, and rather loose 3-D flocs. As a result, the flocs’ sedimentation rate slightly decreases compared to that of individual particles or their small linear aggregates (from 3.5 mm/s for a system without coagulant to 2.5 mm/s for a coagulant concentration of 0.075 wt%). This decrease in sedimentation rate is rather insignificant and is offset by the formation of a denser, more stable precipitate, which effectively resists secondary resuspension, which can significantly reduce the removal effectiveness of microplastic-based water contaminations.

A statistically relevant reduction in the maximum microplastic precipitate volume reaches 14% over 48 hours for a coagulant concentration of 0.1%. This dense and solidified precipitate remains compact and resists secondary resuspension and recontamination of the water after coagulant treatment and settling.

The most effective concentration of BUCOCHEM is 0.05–0.075%, which ensures sufficient precipitate hardening and stabilization with a relatively low coagulant consumption.

Further research in this area is sought to focus on the investigation of the relationship between precipitate compacting and solidification efficiency for other polymer fractions, as well as on expanding the understanding of the mechanism and details of the coagulation process.
