# Supplementary material for: The mechanism and dynamics of the sedimentation of styrene/divinylbenzene microplastics from water and the solidification of the precipitate with an alum-based coagulant “BUCOCHEM”
Source: PLoS One. 2026 Jul 29;21(7):e0346838. doi: 10.1371/journal.pone.0346838 (PMC13421767; doi:10.1371/journal.pone.0346838)
Supplement: Attachment — Submitted filename: Response to Reviewers.doc [file pone.0346838.s002.doc]

Response to Editor/Reviewers

Dear colleagues,

Thank you for reviewing our manuscript. Please find below a complete list of the changes and amendments that have been made following your comments:

1. The revised file has been renamed and uploaded as two copies: a marked-up copy (named as “Revised Manuscript with Track Changes”, all changes highlighted in red) and an unmarked copy (named as “Manuscript”). The entire text of the submission has been revised according to the journal’s templates. Keywords were removed from the manuscript body.
2. We changed our mistaken Data Availability Statement that had been filed at the initial submission of the manuscript. In fact, the manuscript does not report any data, and, therefore, we’ve stated that “The data availability policy is not applicable to this article as it does not report data” in an appropriate section of the Submission form.

Additional Editor’s comments:

Regarding the effect of pH. pH is indeed a key parameter in the coagulation process and the extraction of microplastic particles from water. However, we did not examine the influence of this parameter in our study, as the coagulant under study is intended for use with large volumes of wastewater, where pH correction is typically not performed. Introducing this step into the treatment process unnecessarily complicates it, requiring an additional pH determination, calculation of the required amount of pH-correcting reagent, and a secondary pH determination after its introduction. Given that the pH of municipal wastewater contaminated with microplastics typically ranges from 6.5 to 7.5, the direct addition of the coagulant to the water to be treated and the selection of the most effective dosage are usually considered sufficient and appropriate. Therefore, we did not study the effect of pH on the purification efficiency of the experimental mixture, leaving it at its natural, uncorrected level.

To underline and explain this point, we added a separate paragraph to the end of the Introduction (lines 77-85, marked in red).

Regarding coagulant dosage.

We added a more detailed explanation of the influence of the coagulant concentration on the completeness and easiness of MP removal from water (see Results and Discussion, lines 166-180, marked in red).

Regarding the effect of the sedimentation conditions.

We added a description of the conditions of coagulation and sedimentation of MP. This paragraph should promote a better and clearer understanding of the experimental procedure. It can be found in Materials and Methods (lines 106-111, all marked in red).

Regarding the influence of the water parameters on the process of coagulation and sedimentation.

Since natural waters and municipal wastewaters are complex, multicomponent systems, numerous parameters can affect the coagulation and sedimentation process: the concentration of suspended particles to be coagulated and removed, the presence and concentration of ionic solutes and gases, water temperature, etc. However, in the case of municipal and not heavily contaminated industrial wastewater, their composition is usually not corrected before introducing the coagulant(s). Similarly to the above-mentioned effect of pH, the composition of water is left uncorrected before application of the coagulant. On the other hand, high temperature can significantly deteriorate the efficiency of coagulation since at the temperature of 60 ^o^C or more, the process becomes more unpredictable and can split into local subprocesses, initiating even before complete and uniform distribution of the coagulant across the system is reached. That is why it is advisable to perform preliminary cooling of the water before introducing the coagulant if the water temperature is above 60 ^o^C. This consideration has also been added to the text of our manuscript, in Materials and Methods (lines 112-119, marked in red).

Regarding the kinetics of sedimentation.

Partially, we have discussed this issue in the original version of our manuscript. However, following your comments, we have modified and added more details to explain the influence of the coagulant on the kinetics of sedimentation (lines 211-223, partially marked in red).

We thank you again for your valuable comments and hope than now our modified manuscript can be considered suitable for further editorial processing.

Kind regards,

Igor Winkler, the corresponding author.
