# Supplementary material for: The mechanism and dynamics of the sedimentation of styrene/divinylbenzene microplastics from water and the solidification of the precipitate with an alum-based coagulant “BUCOCHEM”
Source: PLoS One. 2026 Jul 29;21(7):e0346838. doi: 10.1371/journal.pone.0346838 (PMC13421767; doi:10.1371/journal.pone.0346838)
Supplement: Attachment — Submitted filename: Response to Reviewers_R1.doc [file pone.0346838.s003.doc]

Response to Reviewers

Dear colleagues,

Thank you for reviewing our manuscript. Please find below a complete list of the changes and amendments that have been made following your comments.

The revised file has been renamed and uploaded as two copies: a marked-up copy (named “Revised Manuscript with Track Changes,” with all changes highlighted in red) and an unmarked copy (named “Manuscript”).

Additional Reviewers’ comments:

Reviewer #1 hasn’t provided any specific comments and only mentioned some minor corrections that should be made to support the conclusions with data from the Materials and Experimental section & Result and Discussion section.

**Our response**:

We would like to thank Reviewer #1 for his/her efforts in referring our manuscript, and we believe that all changes and amendments made in response to extended comments from Reviewer #2 (see below) will definitely address the point mentioned by Reviewer #1.

Reviewer #2: Title.

**Suggested Revision**: Revise the title to emphasize the coagulation mechanism, floc development, and sediment stabilization rather than simply the application of a commercial coagulant.

**Our Response:**

We agree. The manuscript title and short title have been changed to reflect the matters mentioned in the Reviewer’s comment above.

Abstract

**Suggested Revision**: Clearly state the research gap and objectives. Include essential methodological information. Report quantitative performance indicators. Avoid unsupported mechanistic claims. Include study limitations and practical implications.

**Our response:**

We agree. The abstract has been completely rewritten to pin the points raised by the comment above: we indicated the research gap and objectives of our work clearly, removed some unsupported claims and provided the information about the methodology and limitations of this work. Besides, some potential practical implications are also mentioned in the Abstract.

Introduction

**Suggested Revision**: Explicitly define the research gap and explain how the present study addresses it.

**Our response:**

We added a section (lines 89–96) to the Introduction that further clarifies the research gap addressed in our article. Additionally, we included another reference [10] in the References, and updated the numbering of all subsequent references accordingly.

Literature review

**Suggested Revision:** Expand the discussion of coagulation mechanisms and current challenges in microplastic removal.

**Our response:**

This issue has been partially addressed in the section mentioned above (lines 89-96 in the Introduction). To address your comment, another section of the literature review has been added (lines 97-107), and one more reference [11] was cited. In this fragment, we discuss the influence of flocs' morphology on their ability to resist secondary resuspension and substantiate our assumption of better solidification of branched MP aggregates.

Research Hypothesis:

**Suggested Revision:** Include a clear hypothesis regarding the effects of BUCOCHEM on floc formation, sediment stability, and microplastic removal.

**Our response:**

In fact, a research hypothesis is given in the last paragraph of the Introduction. To strengthen its understanding, we have rephrased that paragraph. Besides, the hypothesis is also explained in the last sentence of the previously added section (see the lines 104-107).

pH Consideration:

**Suggested Revision:** Measure and report initial and final pH values.
Discuss the potential influence of pH on coagulation performance.

**Our response:**

pH is indeed a key parameter in the coagulation process and the extraction of microplastic particles from water, especially when considering coagulation and sedimentation of MP-containing precipitates in various natural aquatic systems. However, we did not examine the influence of this parameter in our study, as the coagulant under study is intended for more ‘technological’use with large volumes of technical or municipal wastewater, where pH correction is typically not performed. Introducing this step into the wastewater treatment process unnecessarily complicates it, requiring an additional pH determination, calculation of the required amount of pH-correcting reagent, and a secondary pH determination after its introduction. Given that the pH of municipal wastewater, whether or not contaminated with microplastics, typically ranges from 5.5 to 7.5, the direct addition of the coagulant to the water to be treated and the selection of the most effective dosage are usually considered sufficient and appropriate. That is why we did not study the effect of pH on the purification efficiency of the experimental mixture, leaving it at its natural, uncorrected level.

To underline and explain this point, we included a separate paragraph at the end of the Introduction (lines 108-116).

Besides, we rewrote the last paragraph of the Introduction to make the above points clearer for potential readers of this article.

Materials and Methods. Water Matrix Selection:

**Suggested Revision**: Validate the results using synthetic wastewater, surface water, or real wastewater samples. Provide water quality characteristics.

**Our response:**

Following your previous comments, we have modified the title and text of our submission to emphasize that this investigation was been primarily focused on issues related to MP removal from not heavily contaminated, mostly industrial wastewater.

Since municipal and industrial wastewaters are complex, multicomponent systems, numerous parameters can affect the coagulation and sedimentation process: the concentration of suspended particles to be coagulated and removed, the presence and concentration of ionic solutes and gases, water temperature, etc. However, in the case of municipal or not heavily contaminated industrial wastewater, their composition is usually not corrected before introducing the coagulant(s), and the results of the experiments on distilled water at some extent can also be extended to the expected efficiency of the coagulation, sedimentation, and solidification of the MP-containing precipitates from real water samples (as long as they are not heavily contaminated).

Microplastic Characterization: Microplastics were characterized only by particle size. No physicochemical characterization was performed. Important properties affecting coagulation behavior remain unknown, including: Surface morphology Surface charge Hydrophobicity Functional groups:

**Suggested Revision** Include SEM, FTIR, contact angle measurements, and zeta potential analysis.

**Our response:**

Based on the aims of this research and available instrumentation, we focused this study on the general properties and stability of the MP precipitates, rather than examining the geometry and morphology of individual particles or separate flocs. Consequently, the specific parameters mentioned in your above comment fell outside the scope of the present work.

Particle Size Selection: The selected particle size (400–500 μm) is considerably larger than many environmentally relevant microplastics. Results may not be representative of smaller microplastics commonly found in aquatic environments. **Suggested Revision**: Investigate multiple particle size ranges or discuss this limitation explicitly.

**Our response:**

We agree. However, as mentioned above, this study primarily focused on particles commonly found in industrial wastewater collected from facilities handling this type of microplastic. Therefore, we examined particles of a size corresponding to the most common fraction in such wastewater. However, in our future studies, we plan to expand this focus to smaller fractions to examine the effectiveness of BUCOCHEM in stabilizing microplastic precipitates formed from smaller fractions. One sentence has been added to the Materials and Experimental Methods (lines 138-140) for better substantiation of this particle size selection.

Coagulant Characterization: BUCOCHEM was characterized only using manufacturer specifications. No independent characterization of the coagulant was conducted. Aluminum speciation and charge characteristics were not evaluated. **Suggested Revision**: Provide independent characterization of the coagulant.

**Our response:**

In this study, we used only one type of commercial coagulant, relying on its manufacturer's specifications. Had its effectiveness in stabilizing microplastic sediment been compared with other products, a separate, independent evaluation would have been conducted. In our case, we meant to test only the effectiveness of this specific product.

Jar Test Procedure: The jar test methodology lacks important operational details. Rapid mixing, slow mixing, mixing intensity (G-value), and standard operating procedures are not adequately described. Reproducibility of the experiments may therefore be limited.

**Suggested Revision:** Follow and report standard jar-test protocols.

**Our response:**

Stirring of the system was performed after adding the required amount of coagulant to achieve uniform distribution. It was short and done using a mechanical mixer with the parameters indicated in the text of our manuscript (line 145). The processes of coagulation, flocculation, sedimentation, and solidification of the precipitate took place in the still, unstirred medium. That is why no additional stirring parameters are required to repeat our experiments.

Statistical Analysis: No inferential statistical analysis was performed. Standard deviations, confidence intervals, and significance testing are absent. The reported optimal dosage is not statistically validated.

**Suggested Revision**: Include ANOVA and appropriate post hoc statistical tests.

**Our response:**

All experiments were repeated 7-10 times, and the results were then averaged. As mentioned in our article, the relative experimental errors were 2.5-3.5 %, which seems quite fair for practical measurements. As seen in Fig. 1, the most effective coagulant concentrations (0.05-0.075 %) cause sediment compaction, greatly exceeding both relative error intervals (for example, compare the sediment layer heights for 0.05% after 6 and 24 h). In fact, this is true even for 0.025% of coagulant; just for 0.05 and 0.075%, the final compactness of the precipitate is better than that for 0.025%. That is why we feel that even without the above-mentioned extended statistical verifications, our results appear relevant.

To emphasize this point and to ensure a better understanding of this conclusion, we added an extra explanation to the Results and Discussion (lines 224-228).

Results: Microplastic Removal Efficiency: The primary objective of the study is microplastic removal; however, removal efficiency was never quantified. Sediment height and settling rate cannot substitute for direct removal measurements. No concentration-based assessment of microplastic removal was performed.

**Suggested Revision**: Quantify microplastic removal efficiency using concentration measurements. Include a complete mass balance.

**Our response:**

Following your valuable comments, we extensively revised the text of our manuscript to clarify that it was mostly aimed at investigating the MP sedimentation mechanism and compacting/stabilizing the precipitate, avoiding its secondary resuspension. The direct examination of the MP removal efficiency was beyond its scope. We hope that this revised article will not mislead potential readers, and it now shows the correct accents and objectives of the study.

Proposed Mechanisms: The concepts of hydrophilization, hydrate layer formation, adsorption, and particle entrapment are repeatedly discussed without direct experimental validation. No analytical evidence is presented to support the proposed mechanisms.

**Suggested Revision**: Verify the proposed mechanisms using SEM, FTIR, EDS, or zeta potential measurements.

**Our response:**

The concept of MP hydrophilization has been thoroughly described in previously published studies, and we cite one such paper [13] in our manuscript. It also includes microphotographs of the hydrophilized surface of microplastic particles. Based on this, and given the available tools and instrumentation, we had to limit ourselves to visual observation of the surface hydrophilization of our plastic particles, which facilitated their more efficient coagulation, aggregation, sedimentation, and compaction. The relevant results and considerations are presented and discussed at the beginning of the Results and Discussion section.

Figure 1: Error bars are absent. Statistical significance between treatments is not reported. Data interpretation remains largely qualitative.

**Suggested Revision**: Include standard deviations and significance testing.

**Our response:**

Error bars are present in Fig. 1, and its caption additionally provides the relative error of our measurements. Statistical relevance of the data is proved by the matters discussed above in the letter (see our response to the comment about ‘Statistical analysis’ above.

Figure 2: Settling behaviour is discussed descriptively without kinetic analysis. No sedimentation model is applied.

**Suggested Revision**: Apply suitable sedimentation or aggregation kinetic models

**Our response:**

Settling behaviour is extensively discussed in the Results and Discussion. We refer to some published results to compare our findings on the dependence of settling rate (shown in Fig. 2) on coagulant concentration as well as flocs shapes and dimensions. This information is necessary to confirm our results and conclusions regarding MP precipitate formation, its stability, and solidification. A more in-depth study of sedimentation kinetics (e.g., the application of certain kinetic sedimentation models and their parameters) was beyond the scope of this study.

Figure 3: Floc morphology is evaluated only visually. No quantitative image analysis is presented.

**Suggested Revision**: Quantify: Floc size, Fractal dimension, Circularity, Aspect ratio using image analysis software.

**Our response:**

This comment is already addressed above in our previous responses (to the ‘Proposed Mechanisms’ and others).

Discussion: The discussion is primarily descriptive and lacks mechanistic depth. The relationship between coagulant dosage and sediment stabilization is not fully explained. Important coagulation mechanisms are not discussed, including: Charge neutralization Sweep flocculation Interparticle bridging Aluminum hydrolysis and speciation Comparisons with conventional coagulants such as alum, PAC, and FeCl₃ are limited.

**Suggested Revision**: Strengthen the mechanistic interpretation and compare findings with recent literature.

**Our response:**

We believe that now, after all modifications of our manuscript made following your valuable comments, its main aim has become more distinct and clear: it reports the efficiency of BUCOCHEM in the formation of a stable and compact precipitate that effectively resists secondary resuspension. Of course, this issue also involves considerations related to floc formation and morphology. The mechanism of coagulation, interparticle interactions, and their kinetics were beyond the scope of our study. That is why we did not examine issues such as zeta potential, charge neutralization, interparticle bridging, and aluminium compound hydrolysis, as they concern the spatial formation and aggregation of particles rather than the formation, compaction, and solidification of the precipitate. Our results and conclusions are discussed and extensively compared to others (references 14-19), most of which were published between 2021 and 2025. The last section of the Results and Discussion explains our hypothesis on better precipitate solidification caused by a more branched and more spatially developed 3-D structure of the MP aggregates. This hypothesis is based on visual microscopic observations and supported by similar results documented in references 19 and 20, published in 2024-2025.

Novelty and Scientific Contribution: The scientific novelty of the study remains limited. Numerous previous studies have already demonstrated microplastic removal using aluminum-based coagulants. The present study mainly confirms the ability of BUCOCHEM to promote floc formation and sediment stabilization. No new mechanistic insight is provided.

**Suggested Revision**: To improve novelty, the authors should consider including: Zeta potential analysis. SEM–EDS characterization of flocs. FTIR investigation of coagulant–microplastic interactions. Kinetic modeling of aggregation and settling. Comparative evaluation against conventional coagulants (PAC, alum, FeCl₃). Optimization using response surface methodology (RSM).

**Our response:**

We generally agree with the points raised in this comment; however, most have already been addressed above. We hope that the revised Abstract and Conclusions now more clearly highlight the novelty and scientific contribution of this work within its current scope.

Conclusion: Several conclusions are stronger than the evidence presented. Claims regarding adsorption, floc architecture, and stabilization mechanisms are not directly demonstrated. The reported optimum dosage is not statistically validated. The limitations of the study are not acknowledged. Future research directions are not provided.

Suggested Revision: Restrict conclusions to experimentally supported findings. Avoid unsupported mechanistic statements. Include study limitations. Recommend future work involving real water matrices and mechanistic characterization.

**Our response:**

In response to your comments, we have completely rewritten this section. We hope that it now more effectively summarizes the main results and findings of this work and outlines possible future directions of its development.

We would like to thank you for your valuable comments and the efforts you put into reviewing our manuscript. Hopefully, it now meets the publication standards and can be considered suitable for further editorial processing.

Kind regards,

Igor Winkler, the corresponding author.
