# Supplementary material for: The mechanism and dynamics of the sedimentation of styrene/divinylbenzene microplastics from water and the solidification of the precipitate with an alum-based coagulant “BUCOCHEM”
Source: PLoS One. 2026 Jul 29;21(7):e0346838. doi: 10.1371/journal.pone.0346838 (PMC13421767; doi:10.1371/journal.pone.0346838)
Supplement: Peer review history

# Editor report: Decision Letter 0

**DOI:** 10.1371/journal.pone.0346838.r001

**Authors:** Muammar Qadafi

31 Mar 2026

PONE-D-26-14340

The Use of an Alum-Based Coagulant “Bucochem” for the Cleaning of Natural Water from Styrene/Divinylbenzene Microplastics

PLOS One

Dear Dr. Winkler,

Thank you for submitting your manuscript to PLOS ONE. After careful consideration, we feel that it has merit but does not fully meet PLOS ONE’s publication criteria as it currently stands. Therefore, we invite you to submit a revised version of the manuscript that addresses the points raised during the review process.

Please submit your revised manuscript by May 15 2026 11:59PM. If you will need more time than this to complete your revisions, please reply to this message or contact the journal office at plosone@plos.org. When you're ready to submit your revision, log on to https://www.editorialmanager.com/pone/ and select the 'Submissions Needing Revision' folder to locate your manuscript file.

Please include the following items when submitting your revised manuscript:

- A letter that responds to each point raised by the academic editor and reviewer(s). You should upload this letter as a separate file labeled 'Response to Reviewers'.
- A marked-up copy of your manuscript that highlights changes made to the original version. You should upload this as a separate file labeled 'Revised Manuscript with Track Changes'.
- An unmarked version of your revised paper without tracked changes. You should upload this as a separate file labeled 'Manuscript'.

If you would like to make changes to your financial disclosure, please include your updated statement in your cover letter. Guidelines for resubmitting your figure files are available below the reviewer comments at the end of this letter.

If applicable, we recommend that you deposit your laboratory protocols in protocols.io to enhance the reproducibility of your results. Protocols.io assigns your protocol its own identifier (DOI) so that it can be cited independently in the future. For instructions see: https://journals.plos.org/plosone/s/submission-guidelines#loc-laboratory-protocols. Additionally, PLOS ONE offers an option for publishing peer-reviewed Lab Protocol articles, which describe protocols hosted on protocols.io. Read more information on sharing protocols at https://plos.org/protocols?utm_medium=editorial-email&utm_source=authorletters&utm_campaign=protocols.

As the corresponding author, your ORCID iD is verified in the submission system and will appear in the published article. PLOS supports the use of ORCID, and we encourage all coauthors to register for an ORCID iD and use it as well. Please encourage your coauthors to verify their ORCID iD within the submission system before final acceptance, as unverified ORCID iDs will not appear in the published article. *Only*  the individual author can complete the verification step; PLOS staff *cannot*  verify ORCID iDs on behalf of authors.

We look forward to receiving your revised manuscript.

Kind regards,

Muammar Qadafi

Academic Editor

PLOS One

Journal requirements:

When submitting your revision, we need you to address these additional requirements.

1. Please ensure that your manuscript meets PLOS ONE's style requirements, including those for file naming. The PLOS ONE style templates can be found at

https://journals.plos.org/plosone/s/file?id=wjVg/PLOSOne_formatting_sample_main_body.pdf and

https://journals.plos.org/plosone/s/file?id=ba62/PLOSOne_formatting_sample_title_authors_affiliations.pdf

2. When completing the data availability statement of the submission form, you indicated that you will make your data available on acceptance. We strongly recommend all authors decide on a data sharing plan before acceptance, as the process can be lengthy and hold up publication timelines. Please note that, though access restrictions are acceptable now, your entire data will need to be made freely accessible if your manuscript is accepted for publication. This policy applies to all data except where public deposition would breach compliance with the protocol approved by your research ethics board. If you are unable to adhere to our open data policy, please kindly revise your statement to explain your reasoning and we will seek the editor's input on an exemption. Please be assured that, once you have provided your new statement, the assessment of your exemption will not hold up the peer review process.

If the reviewer comments include a recommendation to cite specific previously published works, please review and evaluate these publications to determine whether they are relevant and should be cited. There is no requirement to cite these works unless the editor has indicated otherwise.

Additional Editor Comments:

Although the use of an alum-based coagulant (“Bucochem”) for removing styrene/divinylbenzene microplastics from natural water is interesting, the study lacks comprehensiveness. The manuscript would benefit from more detailed investigation of key coagulation parameters, including the effects of pH, coagulant dosage, and sedimentation conditions on removal efficiency. Additionally, the authors should consider incorporating different water matrices or wastewater characteristics, as well as kinetic studies, statistical analysis, and comparisons with other coagulants/studies. Addressing these aspects would significantly strengthen the manuscript and support its suitability for publication.

[Note: HTML markup is below. Please do not edit.]

[NOTE: If reviewer comments were submitted as an attachment file, they will be attached to this email and accessible via the submission site. Please log into your account, locate the manuscript record, and check for the action link "View Attachments". If this link does not appear, there are no attachment files.]

To ensure your figures meet our technical requirements, please review our figure guidelines: https://journals.plos.org/plosone/s/figures

You may also use PLOS’s free figure tool, NAAS, to help you prepare publication quality figures: https://journals.plos.org/plosone/s/figures#loc-tools-for-figure-preparation.

NAAS will assess whether your figures meet our technical requirements by comparing each figure against our figure specifications.

# Author comment: Author response to Decision Letter 1

**DOI:** 10.1371/journal.pone.0346838.r002

21 Apr 2026

Response to Editor/Reviewers

Dear colleagues,

Thank you for reviewing our manuscript. Please find below a complete list of the changes and amendments that have been made following your comments:

1. The revised file has been renamed and uploaded as two copies: a marked-up copy (named as “Revised Manuscript with Track Changes”, all changes highlighted in red) and an unmarked copy (named as “Manuscript”). The entire text of the submission has been revised according to the journal’s templates. Keywords were removed from the manuscript body.

2. We changed our mistaken Data Availability Statement that had been filed at the initial submission of the manuscript. In fact, the manuscript does not report any data, and, therefore, we’ve stated that “The data availability policy is not applicable to this article as it does not report data” in an appropriate section of the Submission form.

Additional Editor’s comments:

Regarding the effect of pH. pH is indeed a key parameter in the coagulation process and the extraction of microplastic particles from water. However, we did not examine the influence of this parameter in our study, as the coagulant under study is intended for use with large volumes of wastewater, where pH correction is typically not performed. Introducing this step into the treatment process unnecessarily complicates it, requiring an additional pH determination, calculation of the required amount of pH-correcting reagent, and a secondary pH determination after its introduction. Given that the pH of municipal wastewater contaminated with microplastics typically ranges from 6.5 to 7.5, the direct addition of the coagulant to the water to be treated and the selection of the most effective dosage are usually considered sufficient and appropriate. Therefore, we did not study the effect of pH on the purification efficiency of the experimental mixture, leaving it at its natural, uncorrected level.

To underline and explain this point, we added a separate paragraph to the end of the Introduction (lines 77-85, marked in red).

Regarding coagulant dosage.

We added a more detailed explanation of the influence of the coagulant concentration on the completeness and easiness of MP removal from water (see Results and Discussion, lines 166-180, marked in red).

Regarding the effect of the sedimentation conditions.

We added a description of the conditions of coagulation and sedimentation of MP. This paragraph should promote a better and clearer understanding of the experimental procedure. It can be found in Materials and Methods (lines 106-111, all marked in red).

Regarding the influence of the water parameters on the process of coagulation and sedimentation.

Since natural waters and municipal wastewaters are complex, multicomponent systems, numerous parameters can affect the coagulation and sedimentation process: the concentration of suspended particles to be coagulated and removed, the presence and concentration of ionic solutes and gases, water temperature, etc. However, in the case of municipal and not heavily contaminated industrial wastewater, their composition is usually not corrected before introducing the coagulant(s). Similarly to the above-mentioned effect of pH, the composition of water is left uncorrected before application of the coagulant. On the other hand, high temperature can significantly deteriorate the efficiency of coagulation since at the temperature of 60 oC or more, the process becomes more unpredictable and can split into local subprocesses, initiating even before complete and uniform distribution of the coagulant across the system is reached. That is why it is advisable to perform preliminary cooling of the water before introducing the coagulant if the water temperature is above 60 oC. This consideration has also been added to the text of our manuscript, in Materials and Methods (lines 112-119, marked in red).

Regarding the kinetics of sedimentation.

Partially, we have discussed this issue in the original version of our manuscript. However, following your comments, we have modified and added more details to explain the influence of the coagulant on the kinetics of sedimentation (lines 211-223, partially marked in red).

We thank you again for your valuable comments and hope than now our modified manuscript can be considered suitable for further editorial processing.

Kind regards,

Igor Winkler, the corresponding author.

Attachment Submitted filename: Response to Reviewers.doc

# Peer review: Decision Letter 1

**DOI:** 10.1371/journal.pone.0346838.r003

**Authors:** Muammar Qadafi

3 Jun 2026

PONE-D-26-14340R1

The Use of an Alum-Based Coagulant “Bucochem” for the Cleaning of Natural Water from Styrene/Divinylbenzene Microplastics

PLOS One

Dear Dr. Winkler,

Thank you for submitting your manuscript to PLOS ONE. After careful consideration, we feel that it has merit but does not fully meet PLOS ONE’s publication criteria as it currently stands. Therefore, we invite you to submit a revised version of the manuscript that addresses the points raised during the review process.

Please submit your revised manuscript by Jul 18 2026 11:59PM. If you will need more time than this to complete your revisions, please reply to this message or contact the journal office at plosone@plos.org. When you're ready to submit your revision, log on to https://www.editorialmanager.com/pone/ and select the 'Submissions Needing Revision' folder to locate your manuscript file.

Please include the following items when submitting your revised manuscript:

- A letter that responds to each point raised by the academic editor and reviewer(s). You should upload this letter as a separate file labeled 'Response to Reviewers'.
- A marked-up copy of your manuscript that highlights changes made to the original version. You should upload this as a separate file labeled 'Revised Manuscript with Track Changes'.
- An unmarked version of your revised paper without tracked changes. You should upload this as a separate file labeled 'Manuscript'.

If you would like to make changes to your financial disclosure, please include your updated statement in your cover letter. Guidelines for resubmitting your figure files are available below the reviewer comments at the end of this letter.

If applicable, we recommend that you deposit your laboratory protocols in protocols.io to enhance the reproducibility of your results. Protocols.io assigns your protocol its own identifier (DOI) so that it can be cited independently in the future. For instructions see: https://journals.plos.org/plosone/s/submission-guidelines#loc-laboratory-protocols. Additionally, PLOS ONE offers an option for publishing peer-reviewed Lab Protocol articles, which describe protocols hosted on protocols.io. Read more information on sharing protocols at https://plos.org/protocols?utm_medium=editorial-email&utm_source=authorletters&utm_campaign=protocols.

As the corresponding author, your ORCID iD is verified in the submission system and will appear in the published article. PLOS supports the use of ORCID, and we encourage all coauthors to register for an ORCID iD and use it as well. Please encourage your coauthors to verify their ORCID iD within the submission system before final acceptance, as unverified ORCID iDs will not appear in the published article. *Only*  the individual author can complete the verification step; PLOS staff *cannot*  verify ORCID iDs on behalf of authors.

We look forward to receiving your revised manuscript.

Kind regards,

Muammar Qadafi

Academic Editor

PLOS One

Journal Requirements:

If the reviewer comments include a recommendation to cite specific previously published works, please review and evaluate these publications to determine whether they are relevant and should be cited. There is no requirement to cite these works unless the editor has indicated otherwise.

[Note: HTML markup is below. Please do not edit.]

Reviewers' comments:

Reviewer's Responses to Questions

**Comments to the Author**

1. If the authors have adequately addressed your comments raised in a previous round of review and you feel that this manuscript is now acceptable for publication, you may indicate that here to bypass the “Comments to the Author” section, enter your conflict of interest statement in the “Confidential to Editor” section, and submit your "Accept" recommendation.

Reviewer #1: (No Response)

Reviewer #2: All comments have been addressed

**********

2. Is the manuscript technically sound, and do the data support the conclusions?

The manuscript must describe a technically sound piece of scientific research with data that supports the conclusions. Experiments must have been conducted rigorously, with appropriate controls, replication, and sample sizes. The conclusions must be drawn appropriately based on the data presented.

Reviewer #1: Partly

Reviewer #2: Partly

**********

3. Has the statistical analysis been performed appropriately and rigorously?

Reviewer #1: Yes

Reviewer #2: Yes

**********

4. Have the authors made all data underlying the findings in their manuscript fully available?

The PLOS Data policy requires authors to make all data underlying the findings described in their manuscript fully available without restriction, with rare exception (please refer to the Data Availability Statement in the manuscript PDF file). The data should be provided as part of the manuscript or its supporting information, or deposited to a public repository. For example, in addition to summary statistics, the data points behind means, medians and variance measures should be available. If there are restrictions on publicly sharing data—e.g. participant privacy or use of data from a third party—those must be specified.

Reviewer #1: Yes

Reviewer #2: Yes

**********

5. Is the manuscript presented in an intelligible fashion and written in standard English?

PLOS ONE does not copyedit accepted manuscripts, so the language in submitted articles must be clear, correct, and unambiguous. Any typographical or grammatical errors should be corrected at revision, so please note any specific errors here.

Reviewer #1: Yes

Reviewer #2: Yes

**********

6. Review Comments to the Author

Please use the space provided to explain your answers to the questions above. You may also include additional comments for the author, including concerns about dual publication, research ethics, or publication ethics. (Please upload your review as an attachment if it exceeds 20,000 characters)

Reviewer #1: The research Paper, "The Use of an Alum-Based Coagulant “Bucochem” for the Cleaning of Natural Water from Styrene/Divinylbenzene Microplastics", is a very good research work but minor correction is needed to support the conclusions with data from Material and experimental section & Result and Discussion section.

Reviewer #2: Title

The title is overly descriptive and resembles a technical report rather than a scientific research article.

The title does not clearly reflect the main scientific contribution of the study.

The phrase “cleaning of natural water” is misleading because all experiments were conducted using distilled water rather than natural water matrices.

The title does not highlight the key investigated phenomena, namely sedimentation behavior, floc formation, and sediment stability.

The novelty of the study is not apparent from the current title.

Suggested Revision:

Revise the title to emphasize the coagulation mechanism, floc development, and sediment stabilization rather than simply the application of a commercial coagulant.

Abstract

The abstract does not clearly identify the research gap that motivated the study.

The study objectives are not explicitly stated.

The experimental design is insufficiently described, making it difficult to understand the scope of the investigation.

Important experimental conditions such as microplastic concentration, coagulant dosage range, number of replicates, and operating conditions are not reported.

No quantitative microplastic removal efficiency is presented.

The study focuses primarily on sediment height reduction and settling behavior rather than actual microplastic removal performance.

Claims regarding adsorption and entrapment of microplastics within coagulant flocs are not supported by direct experimental evidence.

The proposed flocculation mechanism remains speculative.

The limitations of the study are not acknowledged.

Suggested Revision:

Clearly state the research gap and objectives.

Include essential methodological information.

Report quantitative performance indicators.

Avoid unsupported mechanistic claims.

Include study limitations and practical implications.

Introduction

Research Gap:

The introduction provides general information about microplastic pollution and coagulation technologies but fails to clearly identify the specific knowledge gap addressed by this study.

The scientific rationale for selecting BUCOCHEM over other aluminum-based coagulants is not adequately justified.

The novelty of investigating BUCOCHEM for microplastic removal remains unclear.

Suggested Revision:

Explicitly define the research gap and explain how the present study addresses it.

Literature Review:

The literature review is relatively superficial and descriptive.

Recent advances in microplastic coagulation and flocculation mechanisms are not comprehensively discussed.

Most cited studies are used only to provide background information rather than to critically evaluate current knowledge.

The inclusion of more recent studies (2024–2026) would strengthen the scientific context.

Suggested Revision:

Expand the discussion of coagulation mechanisms and current challenges in microplastic removal.

Research Hypothesis:

The manuscript does not present a clear research hypothesis.

The objectives are largely descriptive and do not provide a mechanistic framework for the study.

Suggested Revision:

Include a clear hypothesis regarding the effects of BUCOCHEM on floc formation, sediment stability, and microplastic removal.

pH Consideration:

The justification for excluding pH investigation is scientifically weak.

pH is one of the most important parameters controlling aluminum speciation and coagulation performance.

The absence of pH measurements significantly limits interpretation of the coagulation mechanism.

Suggested Revision:

Measure and report initial and final pH values.

Discuss the potential influence of pH on coagulation performance.

Materials and Methods

Water Matrix Selection:

The study claims applicability to natural water treatment but uses distilled water as the experimental medium.

Distilled water does not adequately represent real environmental or wastewater conditions.

The effects of dissolved organic matter, suspended solids, and ionic strength are not considered.

Suggested Revision:

Validate the results using synthetic wastewater, surface water, or real wastewater samples.

Provide water quality characteristics.

Microplastic Characterization:

Microplastics were characterized only by particle size.

No physicochemical characterization was performed.

Important properties affecting coagulation behavior remain unknown, including:

Surface morphology

Surface charge

Hydrophobicity

Functional groups:

Suggested Revision

Include SEM, FTIR, contact angle measurements, and zeta potential analysis.

Particle Size Selection:

The selected particle size (400–500 μm) is considerably larger than many environmentally relevant microplastics.

Results may not be representative of smaller microplastics commonly found in aquatic environments.

Suggested Revision:

Investigate multiple particle size ranges or discuss this limitation explicitly.

Coagulant Characterization:

BUCOCHEM was characterized only using manufacturer specifications.

No independent characterization of the coagulant was conducted.

Aluminum speciation and charge characteristics were not evaluated.

Suggested Revision:

Provide independent characterization of the coagulant.

Jar Test Procedure:

The jar test methodology lacks important operational details.

Rapid mixing, slow mixing, mixing intensity (G-value), and standard operating procedures are not adequately described.

Reproducibility of the experiments may therefore be limited.

Suggested Revision:

Follow and report standard jar-test protocols.

Statistical Analysis:

No inferential statistical analysis was performed.

Standard deviations, confidence intervals, and significance testing are absent.

The reported optimal dosage is not statistically validated.

Suggested Revision:

Include ANOVA and appropriate post hoc statistical tests.

Results:

Microplastic Removal Efficiency:

The primary objective of the study is microplastic removal; however, removal efficiency was never quantified.

Sediment height and settling rate cannot substitute for direct removal measurements.

No concentration-based assessment of microplastic removal was performed.

Suggested Revision:

Quantify microplastic removal efficiency using concentration measurements.

Include a complete mass balance.

Proposed Mechanisms:

The concepts of hydrophilization, hydrate layer formation, adsorption, and particle entrapment are repeatedly discussed without direct experimental validation.

No analytical evidence is presented to support the proposed mechanisms.

Suggested Revision:

Verify the proposed mechanisms using SEM, FTIR, EDS, or zeta potential measurements.

Figure 1:

Error bars are absent.

Statistical significance between treatments is not reported.

Data interpretation remains largely qualitative.

Suggested Revision:

Include standard deviations and significance testing.

Figure 2:

Settling behavior is discussed descriptively without kinetic analysis.

No sedimentation model is applied.

Suggested Revision:

Apply suitable sedimentation or aggregation kinetic models

Figure 3:

Floc morphology is evaluated only visually.

No quantitative image analysis is presented.

Suggested Revision:

Quantify:Floc size, Fractal dimension, Circularity, Aspect ratio

using image analysis software.

Discussion:

The discussion is primarily descriptive and lacks mechanistic depth.

The relationship between coagulant dosage and sediment stabilization is not fully explained.

Important coagulation mechanisms are not discussed, including:

Charge neutralization

Sweep flocculation

Interparticle bridging

Aluminum hydrolysis and speciation

Comparisons with conventional coagulants such as alum, PAC, and FeCl₃ are limited.

Suggested Revision:

trengthen the mechanistic interpretation and compare findings with recent literature.

Novelty and Scientific Contribution:

The scientific novelty of the study remains limited.

Numerous previous studies have already demonstrated microplastic removal using aluminum-based coagulants.

The present study mainly confirms the ability of BUCOCHEM to promote floc formation and sediment stabilization.

No new mechanistic insight is provided.

Suggested Revision:

To improve novelty, the authors should consider including:

Zeta potential analysis.

SEM–EDS characterization of flocs.

FTIR investigation of coagulant–microplastic interactions.

Kinetic modeling of aggregation and settling.

Comparative evaluation against conventional coagulants (PAC, alum, FeCl₃).

Optimization using response surface methodology (RSM).

Conclusion:

Several conclusions are stronger than the evidence presented.

Claims regarding adsorption, floc architecture, and stabilization mechanisms are not directly demonstrated.

The reported optimum dosage is not statistically validated.

The limitations of the study are not acknowledged.

Future research directions are not provided.

Suggested Revision:

Restrict conclusions to experimentally supported findings.

Avoid unsupported mechanistic statements.

Include study limitations.

Recommend future work involving real water matrices and mechanistic characterization.

**********

7. PLOS authors have the option to publish the peer review history of their article (what does this mean?). If published, this will include your full peer review and any attached files.

If you choose “no”, your identity will remain anonymous but your review may still be made public.

**Do you want your identity to be public for this peer review?**  For information about this choice, including consent withdrawal, please see our Privacy Policy.

Reviewer #1: **Yes:** Tejwant Singh Brar

Reviewer #2: No

**********

[NOTE: If reviewer comments were submitted as an attachment file, they will be attached to this email and accessible via the submission site. Please log into your account, locate the manuscript record, and check for the action link "View Attachments". If this link does not appear, there are no attachment files.]

To ensure your figures meet our technical requirements, please review our figure guidelines: https://journals.plos.org/plosone/s/figures

You may also use PLOS’s free figure tool, NAAS, to help you prepare publication quality figures: https://journals.plos.org/plosone/s/figures#loc-tools-for-figure-preparation.

NAAS will assess whether your figures meet our technical requirements by comparing each figure against our figure specifications.

# Author comment: Author response to Decision Letter 2

**DOI:** 10.1371/journal.pone.0346838.r004

29 Jun 2026

Response to Reviewers

Dear colleagues,

Thank you for reviewing our manuscript. Please find below a complete list of the changes and amendments that have been made following your comments.

The revised file has been renamed and uploaded as two copies: a marked-up copy (named “Revised Manuscript with Track Changes,” with all changes highlighted in red) and an unmarked copy (named “Manuscript”).

Additional Reviewers’ comments:

Reviewer #1 hasn’t provided any specific comments and only mentioned some minor corrections that should be made to support the conclusions with data from the Materials and Experimental section & Result and Discussion section.

Our response:

We would like to thank Reviewer #1 for his/her efforts in referring our manuscript, and we believe that all changes and amendments made in response to extended comments from Reviewer #2 (see below) will definitely address the point mentioned by Reviewer #1.

Reviewer #2: Title.

Suggested Revision: Revise the title to emphasize the coagulation mechanism, floc development, and sediment stabilization rather than simply the application of a commercial coagulant.

Our Response:

We agree. The manuscript title and short title have been changed to reflect the matters mentioned in the Reviewer’s comment above.

Abstract

Suggested Revision: Clearly state the research gap and objectives. Include essential methodological information. Report quantitative performance indicators. Avoid unsupported mechanistic claims. Include study limitations and practical implications.

Our response:

We agree. The abstract has been completely rewritten to pin the points raised by the comment above: we indicated the research gap and objectives of our work clearly, removed some unsupported claims and provided the information about the methodology and limitations of this work. Besides, some potential practical implications are also mentioned in the Abstract.

Introduction

Suggested Revision: Explicitly define the research gap and explain how the present study addresses it.

Our response:

We added a section (lines 89–96) to the Introduction that further clarifies the research gap addressed in our article. Additionally, we included another reference [10] in the References, and updated the numbering of all subsequent references accordingly.

Literature review

Suggested Revision: Expand the discussion of coagulation mechanisms and current challenges in microplastic removal.

Our response:

This issue has been partially addressed in the section mentioned above (lines 89-96 in the Introduction). To address your comment, another section of the literature review has been added (lines 97-107), and one more reference [11] was cited. In this fragment, we discuss the influence of flocs' morphology on their ability to resist secondary resuspension and substantiate our assumption of better solidification of branched MP aggregates.

Research Hypothesis:

Suggested Revision: Include a clear hypothesis regarding the effects of BUCOCHEM on floc formation, sediment stability, and microplastic removal.

Our response:

In fact, a research hypothesis is given in the last paragraph of the Introduction. To strengthen its understanding, we have rephrased that paragraph. Besides, the hypothesis is also explained in the last sentence of the previously added section (see the lines 104-107).

pH Consideration:

Suggested Revision: Measure and report initial and final pH values.

Discuss the potential influence of pH on coagulation performance.

Our response:

pH is indeed a key parameter in the coagulation process and the extraction of microplastic particles from water, especially when considering coagulation and sedimentation of MP-containing precipitates in various natural aquatic systems. However, we did not examine the influence of this parameter in our study, as the coagulant under study is intended for more ‘technological’use with large volumes of technical or municipal wastewater, where pH correction is typically not performed. Introducing this step into the wastewater treatment process unnecessarily complicates it, requiring an additional pH determination, calculation of the required amount of pH-correcting reagent, and a secondary pH determination after its introduction. Given that the pH of municipal wastewater, whether or not contaminated with microplastics, typically ranges from 5.5 to 7.5, the direct addition of the coagulant to the water to be treated and the selection of the most effective dosage are usually considered sufficient and appropriate. That is why we did not study the effect of pH on the purification efficiency of the experimental mixture, leaving it at its natural, uncorrected level.

To underline and explain this point, we included a separate paragraph at the end of the Introduction (lines 108-116).

Besides, we rewrote the last paragraph of the Introduction to make the above points clearer for potential readers of this article.

Materials and Methods. Water Matrix Selection:

Suggested Revision: Validate the results using synthetic wastewater, surface water, or real wastewater samples. Provide water quality characteristics.

Our response:

Following your previous comments, we have modified the title and text of our submission to emphasize that this investigation was been primarily focused on issues related to MP removal from not heavily contaminated, mostly industrial wastewater.

Since municipal and industrial wastewaters are complex, multicomponent systems, numerous parameters can affect the coagulation and sedimentation process: the concentration of suspended particles to be coagulated and removed, the presence and concentration of ionic solutes and gases, water temperature, etc. However, in the case of municipal or not heavily contaminated industrial wastewater, their composition is usually not corrected before introducing the coagulant(s), and the results of the experiments on distilled water at some extent can also be extended to the expected efficiency of the coagulation, sedimentation, and solidification of the MP-containing precipitates from real water samples (as long as they are not heavily contaminated).

Microplastic Characterization: Microplastics were characterized only by particle size. No physicochemical characterization was performed. Important properties affecting coagulation behavior remain unknown, including: Surface morphology Surface charge Hydrophobicity Functional groups:

Suggested Revision Include SEM, FTIR, contact angle measurements, and zeta potential analysis.

Our response:

Based on the aims of this research and available instrumentation, we focused this study on the general properties and stability of the MP precipitates, rather than examining the geometry and morphology of individual particles or separate flocs. Consequently, the specific parameters mentioned in your above comment fell outside the scope of the present work.

Particle Size Selection: The selected particle size (400–500 μm) is considerably larger than many environmentally relevant microplastics. Results may not be representative of smaller microplastics commonly found in aquatic environments. Suggested Revision: Investigate multiple particle size ranges or discuss this limitation explicitly.

Our response:

We agree. However, as mentioned above, this study primarily focused on particles commonly found in industrial wastewater collected from facilities handling this type of microplastic. Therefore, we examined particles of a size corresponding to the most common fraction in such wastewater. However, in our future studies, we plan to expand this focus to smaller fractions to examine the effectiveness of BUCOCHEM in stabilizing microplastic precipitates formed from smaller fractions. One sentence has been added to the Materials and Experimental Methods (lines 138-140) for better substantiation of this particle size selection.

Coagulant Characterization: BUCOCHEM was characterized only using manufacturer specifications. No independent characterization of the coagulant was conducted. Aluminum speciation and charge characteristics were not evaluated. Suggested Revision: Provide independent characterization of the coagulant.

Our response:

In this study, we used only one type of commercial coagulant, relying on its manufacturer's specifications. Had its effectiveness in stabilizing microplastic sediment been compared with other products, a separate, independent evaluation would have been conducted. In our case, we meant to test only the effectiveness of this specific product.

Jar Test Procedure: The jar test methodology lacks important operational details. Rapid mixing, slow mixing, mixing intensity (G-value), and standard operating procedures are not adequately described. Reproducibility of the experiments may therefore be limited.

Suggested Revision: Follow and report standard jar-test protocols.

Our response:

Stirring of the system was performed after adding the required amount of coagulant to achieve uniform distribution. It was short and done using a mechanical mixer with the parameters indicated in the text of our manuscript (line 145). The processes of coagulation, flocculation, sedimentation, and solidification of the precipitate took place in the still, unstirred medium. That is why no additional stirring parameters are required to repeat our experiments.

Statistical Analysis: No inferential statistical analysis was performed. Standard deviations, confidence intervals, and significance testing are absent. The reported optimal dosage is not statistically validated.

Suggested Revision: Include ANOVA and appropriate post hoc statistical tests.

Our response:

All experiments were repeated 7-10 times, and the results were then averaged. As mentioned in our article, the relative experimental errors were 2.5-3.5 %, which seems quite fair for practical measurements. As seen in Fig. 1, the most effective coagulant concentrations (0.05-0.075 %) cause sediment compaction, greatly exceeding both relative error intervals (for example, compare the sediment layer heights for 0.05% after 6 and 24 h). In fact, this is true even for 0.025% of coagulant; just for 0.05 and 0.075%, the final compactness of the precipitate is better than that for 0.025%. That is why we feel that even without the above-mentioned extended statistical verifications, our results appear relevant.

To emphasize this point and to ensure a better understanding of this conclusion, we added an extra explanation to the Results and Discussion (lines 224-228).

Results: Microplastic Removal Efficiency: The primary objective of the study is microplastic removal; however, removal efficiency was never quantified. Sediment height and settling rate cannot substitute for direct removal measurements. No concentration-based assessment of microplastic removal was performed.

Suggested Revision: Quantify microplastic removal efficiency using concentration measurements. Include a complete mass balance.

Our response:

Following your valuable comments, we extensively revised the text of our manuscript to clarify that it was mostly aimed at investigating the MP sedimentation mechanism and compacting/stabilizing the precipitate, avoiding its secondary resuspension. The direct examination of the MP removal efficiency was beyond its scope. We hope that this revised article will not mislead potential readers, and it now shows the correct accents and objectives of the study.

Proposed Mechanisms: The concepts of hydrophilization, hydrate layer formation, adsorption, and particle entrapment are repeatedly discussed without direct experimental validation. No analytical evidence is presented to support the proposed mechanisms.

Suggested Revision: Verify the proposed mechanisms using SEM, FTIR, EDS, or zeta potential measurements.

Our response:

The concept of MP hydrophilization has been thoroughly described in previously published studies, and we cite one such paper [13] in our manuscript. It also includes microphotographs of the hydrophilized surface of microplastic particles. Based on this, and given the available tools and instrumentation, we had to limit ourselves to visual observation of the surface hydrophilization of our plastic particles, which facilitated their more efficient coagulation, aggregation, sedimentation, and compaction. The relevant results and considerations are presented and discussed at the beginning of the Results and Discussion section.

Figure 1: Error bars are absent. Statistical significance between treatments is not reported. Data interpretation remains largely qualitative.

Suggested Revision: Include standard deviations and significance testing.

Our response:

Error bars are present in Fig. 1, and its caption additionally provides the relative error of our measurements. Statistical relevance of the data is proved by the matters discussed above in the letter (see our response to the comment about ‘Statistical analysis’ above.

Figure 2: Settling behaviour is discussed descriptively without kinetic analysis. No sedimentation model is applied.

Suggested Revision: Apply suitable sedimentation or aggregation kinetic models

Our response:

Settling behaviour is extensively discussed in the Results and Discussion. We refer to some published results to compare our findings on the dependence of settling rate (shown in Fig. 2) on coagulant concentration as well as flocs shapes and dimensions. This information is necessary to confirm our results and conclusions regarding MP precipitate formation, its stability, and solidification. A more in-depth study of sedimentation kinetics (e.g., the application of certain kinetic sedimentation models and their parameters) was beyond the scope of this study.

Figure 3: Floc morphology is evaluated only visually. No quantitative image analysis is presented.

Suggested Revision: Quantify: Floc size, Fractal dimension, Circularity, Aspect ratio using image analysis software.

Our response:

This comment is already addressed above in our previous responses (to the ‘Proposed Mechanisms’ and others).

Discussion: The discussion is primarily descriptive and lacks mechanistic depth. The relationship between coagulant dosage and sediment stabilization is not fully explained. Important coagulation mechanisms are not discussed, including: Charge neutralization Sweep flocculation Interparticle bridging Aluminum hydrolysis and speciation Comparisons with conventional coagulants such as alum, PAC, and FeCl₃ are limited.

Suggested Revision: Strengthen the mechanistic interpretation and compare findings with recent literature.

Our response:

We believe that now, after all modifications of our manuscript made following your valuable comments, its main aim has become more distinct and clear: it reports the efficiency of BUCOCHEM in the formation of a stable and compact precipitate that effectively resists secondary resuspension. Of course, this issue also involves considerations related to floc formation and morphology. The mechanism of coagulation, interparticle interactions, and their kinetics were beyond the scope of our study. That is why we did not examine issues such as zeta potential, charge neutralization, interparticle bridging, and aluminium compound hydrolysis, as they concern the spatial formation and aggregation of particles rather than the formation, compaction, and solidification of the precipitate. Our results and conclusions are discussed and extensively compared to others (references 14-19), most of which were published between 2021 and 2025. The last section of the Results and Discussion explains our hypothesis on better precipitate solidification caused by a more branched and more spatially developed 3-D structure of the MP aggregates. This hypothesis is based on visual microscopic observations and supported by similar results documented in references 19 and 20, published in 2024-2025.

Novelty and Scientific Contribution: The scientific novelty of the study remains limited. Numerous previous studies have already demonstrated microplastic removal using aluminum-based coagulants. The present study mainly confirms the ability of BUCOCHEM to promote floc formation and sediment stabilization. No new mechanistic insight is provide

Attachment Submitted filename: Response to Reviewers_R1.doc

# Peer review: Decision Letter 2

**DOI:** 10.1371/journal.pone.0346838.r005

**Authors:** Muammar Qadafi

16 Jul 2026

The mechanism and dynamics of the sedimentation of styrene/divinylbenzene microplastics from water and the solidification of the precipitate with an alum-based coagulant “BUCOCHEM”

PONE-D-26-14340R2

Dear Dr. Winkler,

We’re pleased to inform you that your manuscript has been judged scientifically suitable for publication and will be formally accepted for publication once it meets all outstanding technical requirements.

Within one week, you’ll receive an e-mail detailing the required amendments. When these have been addressed, you’ll receive a formal acceptance letter and your manuscript will be scheduled for publication.

An invoice will be generated when your article is formally accepted. Please note, if your institution has a publishing partnership with PLOS and your article meets the relevant criteria, all or part of your publication costs will be covered. Please make sure your user information is up-to-date by logging into Editorial Manager at Editorial Manager® and clicking the ‘Update My Information' link at the top of the page. For questions related to billing, please contact billing support.

If your institution or institutions have a press office, please notify them about your upcoming paper to help maximize its impact. If they’ll be preparing press materials, please inform our press team as soon as possible -- no later than 48 hours after receiving the formal acceptance. Your manuscript will remain under strict press embargo until 2 pm Eastern Time on the date of publication. For more information, please contact onepress@plos.org.

Kind regards,

Muammar Qadafi

Academic Editor

PLOS One

Additional Editor Comments (optional):

Reviewers' comments:

Reviewer's Responses to Questions

**Comments to the Author**

1. If the authors have adequately addressed your comments raised in a previous round of review and you feel that this manuscript is now acceptable for publication, you may indicate that here to bypass the “Comments to the Author” section, enter your conflict of interest statement in the “Confidential to Editor” section, and submit your "Accept" recommendation.

Reviewer #1: All comments have been addressed

**********

2. Is the manuscript technically sound, and do the data support the conclusions?

The manuscript must describe a technically sound piece of scientific research with data that supports the conclusions. Experiments must have been conducted rigorously, with appropriate controls, replication, and sample sizes. The conclusions must be drawn appropriately based on the data presented.

Reviewer #1: Yes

**********

3. Has the statistical analysis been performed appropriately and rigorously?

Reviewer #1: Yes

**********

4. Have the authors made all data underlying the findings in their manuscript fully available?

The PLOS Data policy requires authors to make all data underlying the findings described in their manuscript fully available without restriction, with rare exception (please refer to the Data Availability Statement in the manuscript PDF file). The data should be provided as part of the manuscript or its supporting information, or deposited to a public repository. For example, in addition to summary statistics, the data points behind means, medians and variance measures should be available. If there are restrictions on publicly sharing data—e.g. participant privacy or use of data from a third party—those must be specified.

Reviewer #1: Yes

**********

5. Is the manuscript presented in an intelligible fashion and written in standard English?

PLOS ONE does not copyedit accepted manuscripts, so the language in submitted articles must be clear, correct, and unambiguous. Any typographical or grammatical errors should be corrected at revision, so please note any specific errors here.

Reviewer #1: Yes

**********

6. Review Comments to the Author

Please use the space provided to explain your answers to the questions above. You may also include additional comments for the author, including concerns about dual publication, research ethics, or publication ethics. (Please upload your review as an attachment if it exceeds 20,000 characters)

Reviewer #1: (No Response)

**********

7. PLOS authors have the option to publish the peer review history of their article (what does this mean?). If published, this will include your full peer review and any attached files.

If you choose “no”, your identity will remain anonymous but your review may still be made public.

**Do you want your identity to be public for this peer review?**  For information about this choice, including consent withdrawal, please see our Privacy Policy.

Reviewer #1: **Yes:** Tejwant SIngh Brar

**********

# Editor report: Acceptance letter

**DOI:** 10.1371/journal.pone.0346838.r006

**Authors:** Muammar Qadafi

PONE-D-26-14340R2

PLOS One

Dear Dr. Winkler,

I'm pleased to inform you that your manuscript has been deemed suitable for publication in PLOS One. Congratulations! Your manuscript is now being handed over to our production team.

At this stage, our production department will prepare your paper for publication. This includes ensuring the following:

* All references, tables, and figures are properly cited

* All relevant supporting information is included in the manuscript submission,

* There are no issues that prevent the paper from being properly typeset

You will receive further instructions from the production team, including instructions on how to review your proof when it is ready. Please keep in mind that we are working through a large volume of accepted articles, so please give us a few days to review your paper and let you know the next and final steps.

Lastly, if your institution or institutions have a press office, please let them know about your upcoming paper now to help maximize its impact. If they'll be preparing press materials, please inform our press team within the next 48 hours. Your manuscript will remain under strict press embargo until 2 pm Eastern Time on the date of publication. For more information, please contact onepress@plos.org.

You will receive an invoice from PLOS for your publication fee after your manuscript has reached the completed accept phase. If you receive an email requesting payment before acceptance or for any other service, this may be a phishing scheme. Learn how to identify phishing emails and protect your accounts at https://explore.plos.org/phishing.

If we can help with anything else, please email us at customercare@plos.org.

Thank you for submitting your work to PLOS ONE and supporting open access.

Kind regards,

PLOS ONE Editorial Office Staff

on behalf of

Dr. Muammar Qadafi

Academic Editor

PLOS One
